# Editorial for the Special Issue “Current Research on Cancer Biology and Therapeutics: Third Edition”

**DOI:** 10.3390/ijms262311537

**Published:** 2025-11-28

**Authors:** Rafael Coveñas

**Affiliations:** 1 Laboratory of Neuroanatomy of the Peptidergic Systems, Institute of Neuroscience of Castilla y León (INCYL), University of Salamanca, c/ Pintor Fernando Gallego 1, 37007 Salamanca, Spain; covenas@usal.es; Tel.: +34-923-294-400 (ext. 1856); 2 Group GIR USAL: BMD (Bases Moleculares del Desarrollo), University of Salamanca, 37007 Salamanca, Spain

In the third edition of this Special Issue, the participating researchers focused their studies on bispecific antibodies against solid tumors [contribution 1], teratoma development [contribution 2], the seminoma microenvironment [contribution 3], neuroblastoma and peptidergic systems [contribution 4], hepatocellular carcinoma [contribution 5], the synthesis of novel podophyllotoxin–benzothiazole congeners for use as anticancer agents [contribution 6], the effects of podophyllotoxin derivatives on non-cancerous diseases [contribution 7], and the involvement of the aryl hydrocarbon receptor and the signal transducer and activator of transcription 3 in chemical carcinogenesis [contribution 8].

Khine Swe Shan’s group from the Division of Hematology and Oncology (Memorial Health Care, Pembroke Pines, FL, USA) has reviewed the use of bispecific antibodies against solid tumors [contribution 1]. These antibodies can simultaneously bind to two different epitopes of the same antigen or different antigens, provided that immune cells kill tumor cells and there is a blockade of the signaling pathways. The bispecific antibodies zenocutuzumab, zanidatamab, tebentafusp, tarlatamab, and amivantamab have been approved by the U.S. Food and Drug Administration for use in treating solid tumors, whereas ivonescimab and cadonilimab have been approved in the People’s Republic of China. In this extensive review, the authors discuss the advances and challenges pertaining to the application of bispecific antibodies to solid tumors; their mechanisms of action, toxicity, and tissue penetration; and resistance therapy. They also report the available data on the bispecific antibodies currently approved in clinical practice and discuss the ones currently being investigated (most of them in early phase I and II trials), targeting, for example, the human epidermal growth factor receptor 2, the prostate-specific membrane antigen, claudin 18.2, the carcinoembryonic antigen, the epithelial cell adhesion molecule, glyican-3, the human leukocyte antigen G, and immune checkpoint molecules. In addition, they establish the future lines of research that should be prioritized [contribution 1], including the combination of bispecific antibody therapies, multi-specific strategies targeting multiple antigens at the same time, or the combination of tyrosine kinase inhibitors or chemotherapy with bispecific antibodies.

Holger M. Reichardt et al. from the Institute for Cellular and Molecular Immunology (University Medical Center Göttingen, Germany) studied the infiltration of immune cells in the 129.MOLF-Chr19 teratoma experimental mouse model [contribution 2]. Teratoma, a differentiated type of testicular germ cell tumor, is the most common solid tumor in young men and is characterized by inflammatory infiltrates. This research group studied the characterization, activation, and polarization state of the infiltrating immune cells in mice that spontaneously develop testicular teratoma (129.MOLF-Chr19) [contribution 2]. The authors reported that testes without alterations in morphology and size can be gathered in several groups depending on the expression of stemness and immune genes; that the neoplastic transformation elicited a first wave of T cell infiltration; and that enlarged tumor testes showed progressive infiltration with macrophages, monocytes, and T and B cells. T cells adopted an inactive state due to the overexpression of immune checkpoint molecules, whereas polarization of macrophages and monocytes to an anti-inflammatory phenotype was reported [contribution 2]. Moreover, the study performed on the metabolic gene expression showed alterations, revealing immune cell infiltration and tumor growth. Thus, in this murine experimental model, T cells were inactivated, favoring the growth of the tumor [contribution 2]. The findings reported in this study will aid in the development of new immunomodulatory therapies against testicular germ cell tumors. Holger M. Reichardt et al. also studied the roles of cell–cell contact and cytokine release in an in vitro model of seminoma microenvironment [contribution 3]. Seminoma, a type II testicular germ cell tumor, is characterized by high infiltration of immune cells. The authors co-cultured purified human T cells or monocytes with the seminoma-derived TCam-2 cell line and found that immune cells were stimulated, because an upregulation of the activation markers and an increased release of pro-inflammatory cytokines (interleukin-6, tumor necrosis factor alpha) were observed when direct physical contact occurred between cells [contribution 3]. Moreover, soluble mediators are involved in favoring a shift in TCam-2 cells from a seminoma-like phenotype to a more dedifferentiated one. The data suggest that pro-inflammatory cytokines outline the tumor microenvironment; that this shift could increase chemoresistance and malignancy in patients suffering from testicular germ cell tumors; and that the cytokines secreted from immune cells are a hopeful therapeutic target for treating this disease [contribution 3].

Manuel Lisardo Sánchez and Rafael Coveñas from the Institute of Neuroscience of Castilla y León (University of Salamanca, Spain) reviewed the involvement of the peptidergic systems in the development of neuroblastoma, the most common childhood extracranial solid tumor, with survival rates below 50% [contribution 4]. Oncogenic peptides such as substance P, vasopressin, angiotensin II, neurotensin, bradykinin, oxytocin, and neuropeptide Y favor the proliferation and migration of neuroblastoma cells as well as angiogenesis, whereas anticancer peptides such as orexin, urocortin, corticotropin-releasing factor, and adrenomedullin counteract all the previous effects [contribution 4]. Accordingly, peptide receptor antagonists (aprepitant, L-733,060, RC-3095, DuP753, tolvaptan, PD123177, H2756, PD123319, and BIIE0246) act as anti-neuroblastoma agents because they block neuroblastoma cell proliferation and migration by inducing apoptosis in tumor cells and angiogenesis [contribution 4]. Other anti-neuroblastoma therapeutic strategies, such as peptide receptor knockdown or the use of focal adhesion kinase or signaling pathway inhibitors, and future research lines in neuroblastoma (combination therapy using chemotherapy/radiotherapy and peptide receptor antagonists, interactions between oncogenic and anticancer peptides, etc.) are also mentioned and discussed [contribution 4]. This review shows the functional complexity of the mechanisms mediated by bioactive peptides in neuroblastoma progression, and the numerous data on this topic suggest that the peptidergic systems are potential and promising targets for the treatment and diagnosis of the disease.

Shih-Ming Huang et al. from the Department of Biochemistry of National Defense Medical Center, Taipei City, Taiwan, reported the chemo-sensitizing actions of 5-fluorouracil- and cisplatin-treated hepatocellular carcinomas induced by lidocaine [contribution 5]. This study was performed using the Hep3B and HepG2 hepatocellular carcinoma cell lines. The combination of the local anesthetic lidocaine with the cytostatics showed that lidocaine acted as a chemosensitizer for both cytostatics in Hep3B/HepG2 cells [contribution 5]. Lidocaine decreased quantities of cytosolic reactive oxygen species, increased the subG1 population, blocked cell proliferation, and promoted autophagy, lipid peroxidation, mitochondrial membrane depolarization, endoplasmic reticulum stress, and apoptosis in both hepatocellular carcinoma cell lines treated with 5-fluorouracil or cisplatin [contribution 5]. Moreover, lidocaine diminished the reduced/oxidized glutathione ratio in HepG2 cells treated with cisplatin or 5-fluorouracil, but this effect was not observed in Hep3B cells [contribution 5]. The authors suggest repurposing lidocaine as an applicable chemosensitizer in the current approach to hepatic artery infusion in chemotherapy procedures, as it could decrease side effects and toxicity in patients suffering from hepatocellular carcinoma. The synergic potential of lidocaine and 5-fluorouracil or cisplatin is important because 90% of the detected liver cancers are diagnosed as hepatocellular carcinomas.

Zbigniew Czarnocki et al. from the Faculty of Chemistry (University of Warsaw, Poland) studied the anticancer properties of newly synthetized podophyllotoxin–benzothiazole derivatives [contribution 6]. After binding to tubulin, podophyllotoxin acts as a tubulin polymerization inhibitor by disrupting microtubule formation, leading to cell cycle arrest. This study was performed using HeLa (human cervical), LOVO (human colorectal), SKOX-3 (human ovarian), MCF-7 (human breast), and B16F10 (mouse melanoma) cancer cell lines [contribution 6]. Two compounds, differing by the presence or absence of an ester group and dubbed 7 and 11 by the authors, exhibited the greatest cytotoxic effects against all the cancer cells studied [contribution 6]. The synthetic pathways of compounds 7 and 11 were also reported; compound 11 was obtained after compound 7 was treated with potassium hydroxide in methanol and neutralized with hydrochloric acid [contribution 6]. Both compounds blocked tumor cell proliferation by promoting G2/M phase arrest in HeLa cells. Moreover, a study of the structure–activity relationship revealed that the free-rotating trimethoxyphenyl group—which binds to the tubulin active site through hydrophobic interactions—and the dioxolane ring are crucial for cytotoxicity [contribution 6]. This study highlights the importance of developing podophyllotoxin–benzothiazole derivatives for use as promising antitumor agents. Izabela Mlynarczuk-Bialy et al. from the Department of Histology and Embryology of Warsaw Medical University, Poland, performed a systematic review on the effects of podophyllotoxin derivatives on non-cancerous diseases [contribution 7]. This angle is important because most of the studies performed on these derivatives have focused on cancer. The authors, following the PRISMA criteria, reported that podophyllotoxin derivatives exert antiviral (against SARS-CoV-2, Dengue, and human papillomavirus), radioprotective, analgesic, antimitotic, and anti-inflammatory effects [contribution 7]. For example, the G-003M derivative exerted a radioprotective effect, and the cyclolignan SAU-22.107 derivative promoted antiviral activity. The promising and widespread therapeutic potential of podophyllotoxin derivatives against non-cancerous diseases is a line of research that should be deepened. Due to its antimitotic effect, podophyllotoxin is currently used for the treatment of genital warts; however, this compound’s toxicity is too high for it to be administered intravenously [contribution 7]. Accordingly, an interesting line of research would be the acquisition of podophyllotoxin derivatives that exhibit lower toxicity. This review highlights the expanding therapeutic potential of podophyllotoxin derivatives beyond cancer. The contributions made by the two Polish research groups highlight the promising application of podophyllotoxin derivatives to treat various diseases, including cancer.

Finally, Pier Giorgio Natali et al. from the Collegium Ramazzini (Bologna, Italy) reviewed the involvement of signal transducer and activator of transcription 3 (STAT3) and the aryl hydrocarbon receptor (AhR) in chemical carcinogenesis [contribution 8]. The authors highlight the essential roles played by AhR (a protein involved in xenobiotic metabolism and detoxification) and the oncoprotein STAT3 (a protein involved in the cell response to environmental pollutant damage) as well as their interplay. Chemicals or their metabolites can trigger irreparable changes in normal cells leading to uncontrolled proliferation and hence converting a normal cell into a tumor cell [contribution 8]. In fact, there has been a significant increase in the number of cancers diagnosed in individuals living in polluted regions; nine million deaths per year are estimated to be due to pollutants [contribution 8]. The ligand-activated transcription factor AhR, which is also involved in cancer maintenance and development, is activated by chemicals like carcinogens, whereas the cytoplasmic transcription factor STAT3 controls immune and inflammatory mechanisms, angiogenesis, apoptosis, and cell proliferation and differentiation, and when it is aberrantly activated, it promotes cancer development by favoring the expression of oncogenic genes [contribution 8]. AhR can interact with the STAT3 pathway (which mediates the carcinogenic effects of some pollutants), and hence chemical agents (hydrocarbon compounds and industrial waste) can alter the STAT3/AhR pathways, thereby favoring the development of cancer [contribution 8]. In this review, the authors highlight that due to environmental pollution’s crucial impact on health, in-depth knowledge of the AhR/STAT3 network is required, and this knowledge could serve to prevent the development of cancer and aid treatment efforts focused on blocking carcinogenesis according to the specific type of pollutant.

In the third edition of this Special Issue, promising antitumor strategies are presented using compounds such as bispecific antibodies [contribution 1], bioactive peptides or peptide receptor antagonists [contribution 4], lidocaine combined with 5-fluorouracil or cisplatin [contribution 5], and podophyllotoxin–benzothiazole compounds [contribution 6]. The current edition includes new studies that deepen our understanding of teratoma development [contribution 2], the seminoma microenvironment [contribution 3], the involvement of the AhR/STAT3 network in chemical carcinogenesis [contribution 8], and the use of podophyllotoxin derivatives to treat several non-cancerous diseases [contribution 7]. All this knowledge must be added to the information on antitumor activity exerted by other compounds mentioned in the first and second editions of this Special Issue (acetylcorynoline, benzo[a]phenoxazine derivatives, sarco/endoplasmic reticulum calcium ATPase inhibitors, neuropeptide Y, neuropeptide Y receptor antagonists, neurokinin-1 receptor antagonists, 4-(dimethylamino) phenyl-5-oxopyrrolidine derivatives, local anesthetics, combination of cisplatin with cannabinoid delta-9-tetrahydrocannabinol, immune checkpoint inhibitors, neurotensin receptor antagonists, somatostatin, methionine-enkephalin, and poly-ADP ribose polymerase inhibitors) and on other anticancer strategies reported in the two previous editions (silencing of the fatty acid elongase 4 and 6 genes, oncolytic vaccinia viruses harboring lectins, and anaphase-promoting complex/cyclosome-cell division cycle 20 activity suppression) to fight teratoma, seminoma, neuroblastoma, hepatocellular carcinoma, melanoma, colorectal cancer, pancreatic ductal adenocarcinoma, papillary thyroid carcinoma, glioma, and breast, prostate, bladder, and ovarian cancer [1,2]. Taken together, the three editions of this Special Issue reveal a plethora of compounds with antitumor effects [3,4,5,6,7,8,9,10,11,12] and deepen our knowledge of various types of cancer, opening promising lines of research. This knowledge will help us explore the anticancer therapeutic strategies reported in the Special Issue, aid in their transfer to clinical practice in the near future, and improve the diagnosis and treatment of patients suffering from cancer.
